# Detection bias and the role of negative control outcomes

**DOI:** 10.1136/bmjmed-2025-001336

**Published:** 2025-06-13

**Authors:** Isaac Núñez, Anthony A Matthews

**Affiliations:** 1 Department of Epidemiology, Harvard T H Chan School of Public Health, Boston, Massachusetts, USA; 2 Unit of Epidemiology, Karolinska Institutet, Stockholm, Sweden

**Keywords:** Epidemiology, Clinical trial

## Abstract

Investigators, patients, or clinicians knowing which treatment is assigned in pragmatic randomised trials and observational analyses can lead to detection bias (ie, systematic differences in determining outcomes between groups). A structural definition of detection bias with directed acyclic graphs is provided, together with several published examples. Why negative control outcomes are best placed to assess detection bias is discussed, and how to correctly select a negative control outcome for this purpose is explained.

**KEY MESSAGES**

- Detection bias arises in unblinded randomised trials and observational analyses when knowing the treatment assigned causes systematic differences in determining outcomes between study groups
- Negative control outcomes, which are outcomes that cannot plausibly be affected by the treatment, can help to assess detection bias
- An appropriate negative control outcome, however, must share the same unmeasured determinants of ascertainment as the outcome of interest

## Introduction

Pragmatic randomised trials and observational analyses that emulate pragmatic trials (ie, target trial emulations) are increasingly used to assess the effectiveness and safety of treatments.^1^,^3^ A core concept of both study designs, regardless of randomisation, is unblinded (open label) treatment assignment. In pragmatic trials, one motivation for not blinding the treatment assigned is that the resulting evidence reflects real world conditions where patients and clinicians are aware of the treatment they are given. Also, some interventions (eg, lifestyle or surgical interventions) might not be amenable to blinding. By definition, observational analyses cannot be blinded.

If investigators, patients, or treating clinicians know which treatment is assigned, in both trials and observational studies, this knowledge can lead to bias because of differences in determining outcomes, so called detection bias.^4 5^ Although this bias has been acknowledged, little guidance exists on approaches that can help to understand the extent of the problem.^4^,^6^ Here, we revisit the definition of detection bias, provide published examples of its occurrence, and explain how negative control outcomes can be used to assess the presence of detection bias.

## Defining detection bias

Detection bias arises from systematic differences in determining outcomes between study groups, which is influenced by knowing which treatment is assigned.^4^ When studies lack blinding, for example, in unblinded pragmatic trials or observational analyses, detection bias is a concern.

Patients, healthcare providers, or investigators knowing which treatment is assigned can affect outcome ascertainment in different ways. Based on the treatment received, patients might seek more frequent care, healthcare providers might monitor patients more closely, and healthcare providers or investigators, or both, might be more likely to increase the length of follow-up or ask probing questions. Detection bias is more often the result of an expected and appropriate change in behaviour by healthcare providers and patients in response to a known treatment. This finding contrasts with performance bias, where the quality of care is different between treatment arms and possibly results from bad or unethical research practices.^5^ Performance bias can occur, for example, in the per-protocol analysis of an unblinded trial where healthcare providers are stakeholders in the study and thus have a personal interest in the success of the evaluated treatment, resulting in additional interventions being performed in participants in their favoured study arm.

All outcomes, except all cause mortality, can be influenced by detection bias to some degree. Table 1 lists some clinical outcomes with specific examples, ordered by their proposed likelihood of having detection bias. The method used to collect outcomes also influences the possibility of detection bias. Questionnaires and telephone calls by investigators are prone to recall or probing questions, which can easily be modified by knowing the treatment because the outcomes are collected within the context of the study. In contrast, outcomes collected from routine data sources, such as electronic health records or registers, are less likely to be influenced by participation in the study. In this case, differences in the recording of outcomes do not depend on the investigators, but require a patient or healthcare provider to actively modify their behaviour as a consequence of the assigned treatment.

**Table 1:** Example of outcomes and their perceived likelihood of having detection bias

Likelihood of having detection bias	Type of outcome	Examples
Least likely	All cause mortality	—
	Cause specific mortality	Death from myocardial infarction, stroke, or pneumonia
	Severely symptomatic life threatening outcome	Major stroke, myocardial infarction, major bleeding, or major traumatic fractures
	Severely symptomatic non-life threatening outcome	Non-major bleeding, hallucinations, or minor traumatic fractures
	Asymptomatic outcome	Cholesterol levels, early chronic kidney disease, early type 2 diabetes, or most instances of hypertension
	Symptomatic non-life threatening outcome	Constipation, skin rash, or uncontrolled type 2 diabetes
Most likely	Subjective outcomes reported by patient	Anxiety, headache, fatigue, or mild non-specific pain

Detection bias can be exemplified with a pragmatic trial evaluating starting versus not starting anticoagulation treatment in survivors of a major stroke. If the outcome is a secondary stroke, its severity and symptoms make it difficult to miss, regardless of the study group or the method used to collect outcomes (eg, health records or questionnaires). Any differences in determining the outcome between groups are likely negligible. If the outcome is a headache, however, higher reporting in those assigned to anticoagulation treatment is possible because this group is monitored more closely by healthcare providers. An outcome such as a transient ischaemic attack might fall somewhere in between. Also, an outcome can be in different levels (based on the levels listed in table 1) depending on its definition. A diagnosis of asymptomatic diabetes (during routine screening) is more likely to be affected by detection bias than symptomatic diabetes (testing for weight loss, polyuria, and excessive thirst), and diabetes diagnosed during a hyperglycaemic crisis will almost always be identified correctly.

**Figure 1:**
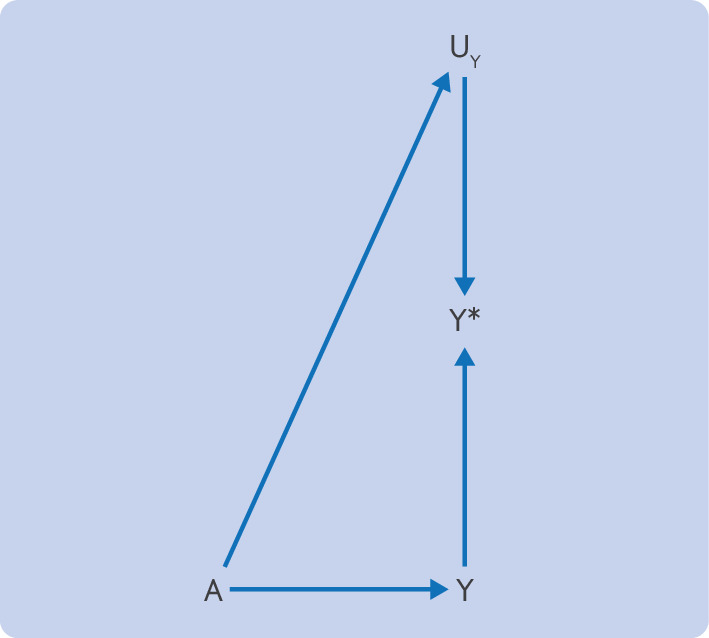
Detection bias. The treatment, A, directly affects the true outcome, Y, but also the ascertained outcome (Y*), through unmeasured determinants of detection (U_Y_). Here, U_Y_ represents sources of detection bias such as an individual's healthcare seeking behaviour and the follow-up established by a clinician for that individual

Figure 1 shows the structure of detection bias with a directed acyclic graph.^7^,^9^ The aim is to estimate the effect of an assigned treatment, A, on the outcome, Y. Our data, however, only provide a measure of the outcome, Y*. Assume the data are from an open label randomised trial, such that no common causes of A and Y (ie, no confounding) exist. If any unmeasured determinants of the measured outcome, U_Y_, that are affected by the treatment exist, such as propensity to seek healthcare, the estimated effect of treatment on the measured outcome will be a combination of both its effect on the outcome and its effect on the unmeasured determinants of ascertainment. Thus U_Y_ is a mediator of the effect of the intervention on the measured outcome Y*.

## Examples of detection bias

We provide three published examples of detection bias: in a randomised trial for a symptomatic outcome; in an observational study for an outcome that can be symptomatic or asymptomatic; and in an observational study for an asymptomatic outcome.

### Surgical masks and respiratory infection

**Figure 2:**
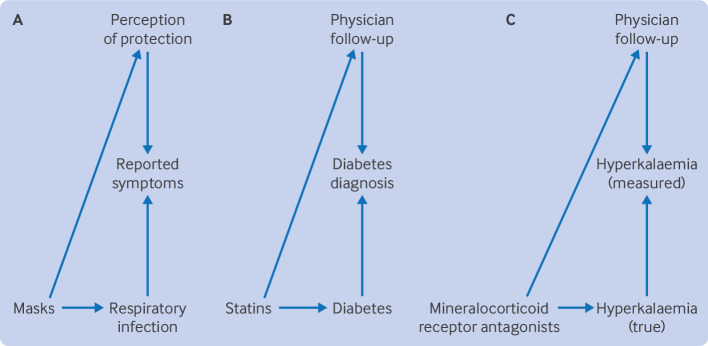
Published examples likely having detection bias. (**A**) Surgical masks and respiratory infection. (**B**) Statins and diabetes. (**C**) Stopping treatment with mineralocorticoid receptor antagonists and hyperkalaemia. Common causes of treatment and outcome are omitted in the observational studies to avoid clutter

A pragmatic trial evaluated the effectiveness of using surgical masks in public to reduce respiratory infections.^10^ Participants were randomised to mask in public, and the main outcome was determined with a questionnaire about symptoms after 14 days. Investigators had no knowledge of the intervention, but trial participants were clearly aware of their assigned group. Wearing a mask is an intervention for which its perceived effectiveness differs according to who uses it, particularly since the covid-19 pandemic. As such, wearing a mask likely affected the healthcare seeking behaviour of participants in the trial and the likelihood of reporting symptoms in the questionnaire (figure 2A). For example, those participants randomised to wear a mask could be less likely to attribute non-specific symptoms to a respiratory infection because they felt more protected. This finding could partly explain the lower odds of respiratory symptoms reported in people who were randomised to wear masks in the trial, particularly because the largest benefit was found in the subgroup that at baseline believed masks were beneficial.

### Statins and diabetes

An observational analysis that emulated a target trial estimated an increased risk of diabetes in people who started taking statins compared with those who did not take statins.^11^ Individuals who started statins could have been more likely to seek healthcare because of attributing new symptoms to the treatment or to healthcare providers giving more appointments to monitor the drug, which increased the opportunities diabetes screening (figure 2B). The likelihood of a diagnosis of diabetes could, therefore, be artificially higher in those who started statins, which could partly explain the increased risk estimated in the study.

### Stopping mineralocorticoid receptor antagonists and hyperkalaemia

Another target trial emulation evaluated stopping versus continuing treatment with mineralocorticoid receptor antagonists (a drug treatment that increases levels of potassium as a side effect) in people with chronic kidney disease and raised levels of blood potassium (ie, hyperkalaemia).^12^ Outcomes included incident cardiovascular disease or death and recurrent hyperkalaemia.^12^ Detection bias might partly explain the higher risk of recurrent hyperkalaemia in the group that continued to take mineralocorticoid receptor antagonists. To be eligible for the study, individuals must have had hyperkalaemia, possibly as a consequence of taking mineralocorticoid receptor antagonists (but not exclusively so); hence individuals that continued to use this drug would undergo more regular routine testing. Given that hyperkalaemia is asymptomatic unless extreme, more testing leads to a higher probability of detecting recurrent hyperkalaemia even if no true difference exists (figure 2C).

## Negative control outcomes to assess detection bias

We have described detection bias and given clear examples. We will now explain how we can choose an appropriate negative control outcome to assess the presence of detection bias. A negative control outcome is one on which the treatment under study has no plausible effect or the effect is known to be null. Hence an association between the treatment and the negative control outcome in the data must be a consequence of bias.^13^ Negative control outcomes are generally not bias specific, and any association must be interpreted carefully in the context of the study.^13^,^16^ For instance, in the observational studies described in the previous section, a competing concern is residual confounding (ie, unaccounted common causes of A and Y). Residual confounding would also be a concern when estimating the per protocol effect, but not when estimating the intention-to-treat effect in the example of the randomised trial.^17^

**Figure 3:**
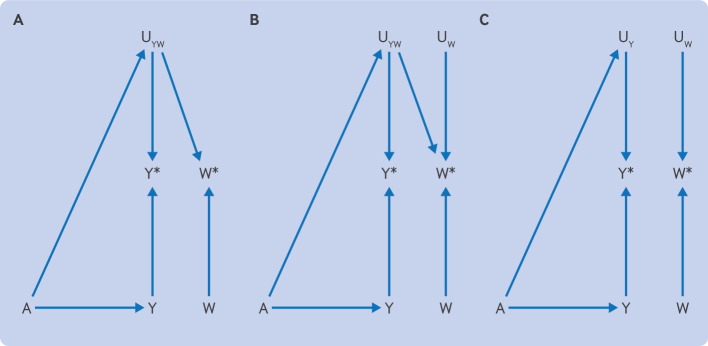
Negative control outcomes for detection bias. (**A**) Example of the use of an appropriate negative control outcome. The ascertained outcome, Y*, and the ascertained negative control outcome, W*, are both affected by the same determinants of detection (U_YW_). Given that treatment A does not affect the negative control outcome, W, other than through U_YW_, any observed association is expected to be due to U_YW_. If an association is observed between A and Y* but not between A and W*, then U_YW_ is negligible. (**B, C**) Examples of where a suboptimal negative control outcome was selected. (**B**) W* shares some but not all determinants of detection with Y*. Depending on the context, the determinants of detection that are not shared (U_W_) might make W* ineffective as a negative control outcome. (**C**) Y* and W* do not share determinants of detection, and consequently no path exists from A to W*. Thus the negative control outcome does not provide information about the possibility of detection bias, and any result will only be misleading

When selecting an appropriate negative control outcome to assess detection bias, it must share the same unmeasured determinants of ascertainment with the outcome of interest.^13 18^ The directed acyclic graph in figure 3 illustrates the structure of an optimally (figure 3A), suboptimally (figure 3B), and incorrectly (figure 3C) selected negative control outcome to assess detection bias.

Figure 3A shows that to be an appropriate choice of negative control to assess detection bias, the determinants of detection (U_YW_) must be shared by the measured negative control outcome (W*) and the measured outcome of interest (Y*). If more determinants of detection of the measured negative control outcome (U_W_) exist, the bias will be incompletely accounted for (figure 3B) or will be completely ignored (figure 3C). Ideally, the negative control outcome will have characteristics, such as symptomatology, diagnostic work-up, severity, and other determinants of detection, aligned with the main outcome.

Taking the example of statins and diabetes, the investigators used peptic ulcers as a negative control outcome.^11^ Peptic ulcer is generally a symptom based diagnosis (ie, the diagnosis usually occurs after an individual seeks care for abdominal pain, nausea and vomiting, or blood in stools, for example). Diabetes, however, is often asymptomatic, and a diagnosis often occurs after screening in individuals with risk factors, such as obesity or a family history. Figure 3B depicts this scenario; some of the determinants of the measured negative control and measured outcomes are the same (if both have symptoms of similar severity), but some are independent. Thus peptic ulcer is likely an appropriate negative control outcome for symptomatic diabetes but is suboptimal for asymptomatic diabetes. If the outcome included all diabetes diagnoses, peptic ulcer would be only partially effective as a negative control outcome (figure 3B).

In the example of stopping versus continuing mineralocorticoid receptor antagonists and hyperkalaemia, bone fractures were used as a negative control.^12^ Hyperkalaemia is asymptomatic and identified from blood measurements, whereas fractures are generally symptomatic and emergency care is often required. Figure 3C largely depicts this scenario. The unmeasured determinants of ascertainment of the measured negative control and measured outcomes are independent. A more appropriate negative control outcome might be another laboratory value, such as cholesterol or liver function tests, where mineralocorticoid receptor antagonists have no plausible effect, but would likely also be tested in individuals that use mineralocorticoid receptor antagonists (eg, people with heart failure). In both of these scenarios, the main outcome and the negative control do not share the same determinants of ascertainment, so detection bias cannot be ruled out.

## Choice of negative control outcome to assess detection bias

A negative control outcome used to assess detection bias must be determined comparably with the outcome of interest. Being in the same level as shown in table 1 increases the likelihood of sharing determinants of ascertainment. For example, if the outcome of interest is major stroke which is symptomatic and life threatening, a good choice of negative control outcome might be major traumatic fractures (if no plausible effect of the treatment on the risk of fractures exists). Both require immediate hospital care. Therefore, both outcomes have a similar likelihood of being reported and recorded, regardless of whether the outcomes were measured by questionnaires, medical records, or any other mechanism. If there is no association between the treatment and fractures, then detection bias is likely absent or negligible.

Selecting an appropriate negative control outcome might be more challenging for less severe outcomes, because determining if two outcomes are at a similar level (based on levels in table 1) is more nuanced. For example, if the outcome of interest is symptomatic covid-19 (eg, when evaluating the effectiveness of covid-19 vaccines), an appropriate negative control would be symptomatic influenza or other symptomatic respiratory infection. Both outcomes have overlapping symptoms of fever, cough, and fatigue, for example, and have coinciding diagnostic pathways. If an individual seeks care because of these symptoms, which might be more likely if they have received a specific treatment, conceivably they will be tested for both covid-19 and influenza (unless, for example, the symptoms occurred during the peak of the covid-19 pandemic). So if an estimated effect of a treatment on covid-19 but not influenza exists, again a reasonable assumption is that the effect on covid-19 is a consequence of treatment, not a difference in a tendency to seek or receive a test for covid-19 between treatment groups. Hypertension would likely not be a good choice of negative control outcome for covid-19. Determinants of receiving a covid-19 test (eg, symptoms of covid-19) are different from determinants of having a blood pressure measurement.

An alternative negative control outcome could be the main outcome but determined during a period where no plausible effect exists. The advantage is that the determinants of detection are known to be the same. For example, observational studies have compared the risk of covid-19 among individuals who were vaccinated and not vaccinated during the two weeks after vaccination.^19^ Because antibodies have not yet formed, any differential risk during this time likely represents residual bias (eg, if individuals who were vaccinated were more likely to be aware and report any covid-19 symptoms).

**Figure 4:**
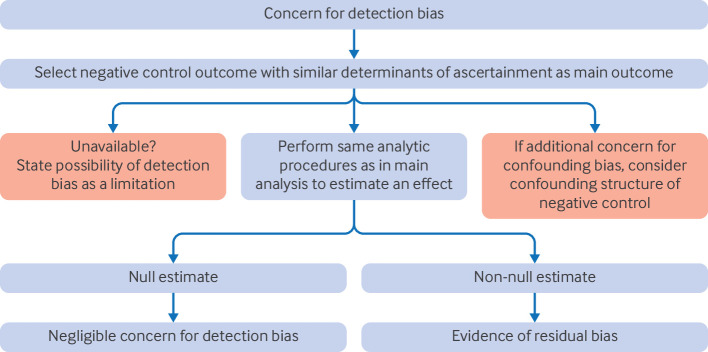
Considerations when assessing the presence of detection bias with negative control outcomes

The negative control outcome must be reasonably frequent because low precision could complicate the interpretation of the negative control analysis.^16 20^ No formal guidance exists on how frequent a negative control outcome must be, and therefore a judgment call, based on subject matter knowledge, is required.^13 16 20 21^ If the negative control analysis was prespecified, then it should be performed while acknowledging low precision as a limitation. Figure 4 outlines practical considerations when assessing detection bias with negative control outcomes.

## Limitations of negative control outcomes to assess detection bias

**Figure 5:**
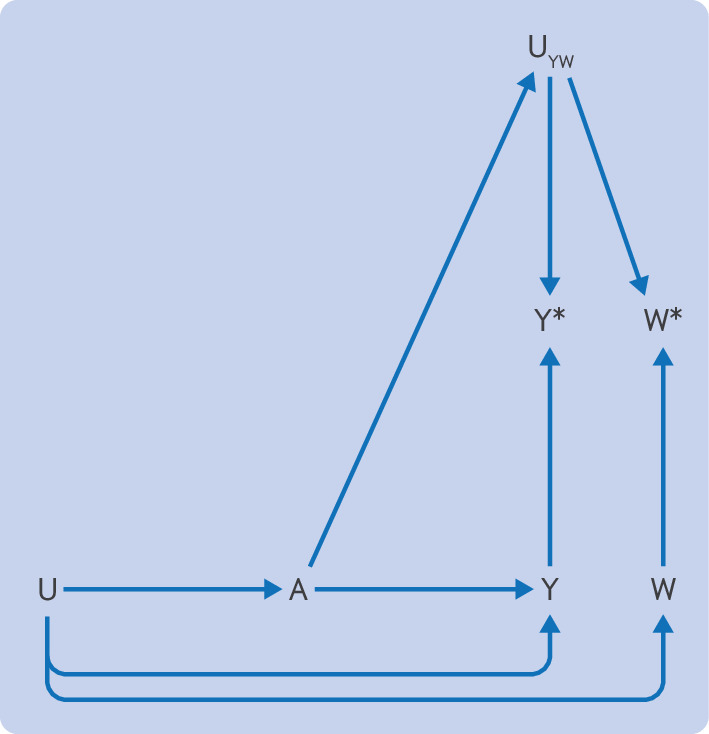
Coexistence of confounding and detection bias. U=unmeasured confounders, A=treatment, Y=true outcome, Y*=ascertained outcome, W=negative control outcome, W*=ascertained negative control outcome, U_YW_=determinants of detection

Several caveats exist on the use of negative control outcomes to assess detection bias. Firstly, even if the perfect negative control outcome is chosen, attributing any association between the treatment and the negative control outcome to detection bias requires that this be the primary suspected source of bias. Other sources of bias (eg, confounding bias) should be secondary concerns. For example, figure 5 depicts a scenario where detection bias coexists with unmeasured confounding (U). This scenario could be an observational study where insufficient adjustment for confounding might be found.^20 22^ Although an inverse association exists between the severity of the outcome and detection bias, the relationship between outcome severity and confounding is likely monotonic; that is, more severe outcomes, such as death or cancer, are likely more complex and affected by more U variables (figure 5) and thus more confounded.

Secondly, the appropriate choice of negative control outcome should ideally be based on data that empirically show that the treatment has a negligible effect. This approach might be challenging when evaluating new treatments, which could have many unknown effects (eg, glucagon-like peptide 1 agonists, sodium-glucose co-transporter 2 inhibitors, or new antiretroviral agents). The justification for selecting a particular negative control outcome should be clearly stated, as well as any references supporting the choice.

Thirdly, negative control outcomes might be available that are not at the same level as the main outcome in table 1 but still share some determinants of detection (as in figure 3B). In these instances, including the negative control outcome could be reasonable if it is acknowledged and discussed that these are not perfectly comparable outcomes. These negative control outcomes could be useful if an association is found (indicating the presence of detection bias), but lack of an association does not rule out detection bias.

Fourthly, negative control outcomes might not be available in the study dataset, or the available outcomes could be too different from the main outcome (as in figure 3C) such that important determinants of detection are unlikely to be shared. If possible, blinding should be incorporated during the planning stage of a pragmatic trial if detection bias is a concern.^23^ Alternatively, a negative control analysis could be discussed and included during the planning stage. In an observational analysis with pre-existing data, the possibility of detection bias should be acknowledged and stated as a limitation when a convincing negative control outcome is unavailable.

## Conclusions

Detection bias can be a problem in pragmatic trials and their observational emulations because non-blinded treatment assignment can affect healthcare seeking behaviours and medical practices which, in turn, can influence the likelihood of identifying an outcome. The notable exception is the outcome of all cause mortality. Negative control outcomes are a valid way to assess if detection bias is a major problem in any given study. Negative control outcomes must reasonably share the same determinants of detection as the outcome of interest, however, because otherwise a finding of no association between the treatment and negative control outcome does not exclude detection bias for the outcome of interest.

## Data Availability

No data are available.
