# Supplementary material for: Detection bias and the role of negative control outcomes
Source: BMJ Med. 2025 Jun 13;4(1):e001336. doi: 10.1136/bmjmed-2025-001336 (PMC12172073; doi:10.1136/bmjmed-2025-001336)
Supplement: Supplementary file 1 [file bmjmed-2025-001336.reviewer_comments.pdf]

PEER REVIEW HISTORY

BMJ Medicine publishes all reviews undertaken for accepted manuscripts. Reviewers are asked to complete a checklist review form and are provided with free text boxes to elaborate on their assessment. These free text comments are reproduced below.

ARTICLE DETAILS

**TITLE (PROVISIONAL)**	Detection bias and the role of negative control outcomes
**AUTHORS**	Núñez, Isaac; Matthews, Anthony

VERSION 1 - REVIEW

**REVIEWER NAME**	Davies, Neil
**REVIEWER AFFILIATION**	UCL, Division of Psychiatry and Department of Statistical Science
**REVIEWER CONFLICT OF INTEREST**	None
**AI DISCLOSURE**	No
**DATE REVIEW RETURNED**	07-Mar-2025

**GENERAL COMMENTS**	bmjmed-2025-001336: Detection bias and the role of negative control outcomes<br>This is an interesting study that describes how to use negative control outcomes to assess detection bias. This is a common problem in experimental and observational studies. The paper reads well and is correct – I have no substantive methodological comments. The biggest challenge faced by this paper is selecting a negative control outcome and demonstrating or evaluating if it is valid. The authors cover this point in some detail and acknowledge the limitations and challenges of this approach. Thus, this would make a nice contribution to the methodological literature and covers ideas not covered elsewhere.<br>I hope my minor comments below improve the paper and thank you for writing and submitting it.<br><br>Minor<br>“U-comparable” is a bit of jargon you could probably drop for this audience. The key point (as I understand) is that your -ve control outcome must have a similar confounding structure as your outcome of interest. You define it as “such that symptomatology, diagnostic workup, severity, and other determinants of detection are aligned, but treatment still has no plausible or known effect on the negative control outcome.” But this is somewhat hidden in the text. So either you could define this term more explicitly or explain without this term.<br><br>“Furthermore, the negative control outcome must occur with reasonable frequency. If it is rare, little precision may erroneously lead to a conclusion of no association.” This is pretty vague. Is it possible to provide more specific guidance about statistical power here? A key challenge is that a negative result (i.e. failing to detect an association) is, in this case, anti-conservative, and understanding when a negative control analysis is likely to be sufficiently powered would be essential for using this approach.<br><br>Minor

	Piccininni and Stensrud have a nice paper on negative control populations, which might be worth citing, but up to you, as this is clearly a very different methodological problem (negative control outcomes).<br><br>https://pubmed.ncbi.nlm.nih.gov/38465949/<br><br>Line 230 – typo . .

VERSION 1 – AUTHOR RESPONSE

Reviewer: 1

C: This is an interesting study that describes how to use negative control outcomes to assess detection bias. This is a common problem in experimental and observational studies. The paper reads well and is correct – I have no substantive methodological comments. The biggest challenge faced by this paper is selecting a negative control outcome and demonstrating or evaluating if it is valid. The authors cover this point in some detail and acknowledge the limitations and challenges of this approach. Thus, this would make a nice contribution to the methodological literature and covers ideas not covered elsewhere.
I hope my minor comments below improve the paper and thank you for writing and submitting it.
R: We appreciate your comments and time.

C: Minor
"U-comparable" is a bit of jargon you could probably drop for this audience. The key point (as I understand) is that your -ve control outcome must have a similar confounding structure as your outcome of interest. You define it as "such that symptomatology, diagnostic workup, severity, and other determinants of detection are aligned, but treatment still has no plausible or known effect on the negative control outcome." But this is somewhat hidden in the text. So either you could define this term more explicitly or explain without this term.
R: We have dropped the term because it is confusing. Typically, papers on negative controls have focused on confounding, in which case "U-comparable" means having that a negative control outcome has a similar confounding structure than the main outcome. In this case, we had referred to "U-comparable" as having the same unmeasured determinants of ascertainment, which are mediators. Thus, the term resulted confusing, so we opted to drop it.
In addition, we have added several phrases to make an explicit distinction between detection bias and confounding, as well as Figure 5 which shows a structure in which they may coexist. The phrases now read:
• Lines 115-117: "However, our data only provide a measure of the outcome, Y*.Assume the data comes from an open label randomized trial, such that there are no common causes of A and Y (i.e. no confounding)."
• Lines 172-178: "Negative control outcomes are generally not bias-specific, and any association must be interpreted carefully in the context of the study..17–20 For instance, in the observational studies described in the previous section a competing concern is residual confounding (i.e .unaccounted common causes of A and Y). This would not be a concern, however, in the example of the randomized trial.21"
• Lines 261-268: "Other sources of bias (e.g. confounding bias) should be secondary concerns. For example, Figure 5 depicts a scenario in which selection bias coexists with unmeasured confounding (U). This could be the case in an observational study where there might be insufficient confounding adjustment.22,23 However, while there is an inverse association between outcome severity and detection bias, the association between outcome severity and confounding is likely monotonic. That is, more severe outcomes, such as death or cancer, are likely affected by more complex and affected

by more U variables (Figure 5), and thus more confounded.”

C: “Furthermore, the negative control outcome must occur with reasonable frequency. If it is rare, little precision may erroneously lead to a conclusion of no association.” This is pretty vague. Is it possible to provide more specific guidance about statistical power here? A key challenge is that a negative result (i.e. failing to detect an association) is, in this case, anti-conservative, and understanding when a negative control analysis is likely to be sufficiently powered would be essential for using this approach.
R: We opted to remove any mention of the required frequency of the negative control outcomes to avoid confusion or ambiguity. No formal guidance exists, and other papers that deal with negative control outcomes (with a focus on confounding bias) just recommend a “reasonably frequent” negative control outcome (e.g. DOI: 10.1002/pds.5623, DOI: 10.1007/s40471-020-00243-4). Because of this, we found that any mention about the required frequency would introduce questions, rather than solving them.

C: Piccininni and Stensrud have a nice paper on negative control populations, which might be worth citing, but up to you, as this is clearly a very different methodological problem (negative control outcomes).
https://pubmed.ncbi.nlm.nih.gov/38465949/
R: We enjoyed the read; however we did not find a way to naturally incorporate the reference.

C: Line 230 – typo . .
R: We fixed the typo.
